# Articulatory suppression during instruction encoding impedes performance in choice reaction time tasks

**DOI:** 10.3758/s13423-022-02100-5

**Published:** 2022-05-06

**Authors:** Félice van ’t Wout, Christopher Jarrold

**Affiliations:** 1 School of Psychology, College of Life and Environmental Sciences, University of Exeter, Exeter, UK; 2 School of Psychological Science, University of Bristol, Bristol, UK

**Keywords:** Skill acquisition, Instruction following, Language, Learning

## Abstract

Theories of instruction following assume that language contributes to our ability to understand and implement instructions. The two experiments reported here investigated that assumption. Participants (total N = 96) were required to learn a series of novel tasks, with each task consisting of six arbitrary stimulus-response rules. All tasks were preceded by an instruction phase (a visual depiction of the correct stimulus-response rules for each task), during which participants performed a verbal distractor task (articulatory suppression), a non-verbal distractor task (foot tapping) or no distractor task. Additionally, the duration of the instruction phase was varied so that it was either long (60 s) or short (30 s in Experiment 1, or 10 s in Experiment 2). In both experiments participants made more errors when they had performed articulatory suppression during the instruction interval, compared to the foot tapping and no distractor task conditions. Furthermore, Experiment 2 found that this detrimental effect of articulatory suppression was especially pronounced with a very short instruction duration. These findings demonstrate that language plays a crucial role in the encoding of novel task instructions, especially when instructions are encoded under time pressure.

## Introduction

The ability to understand and implement instructions is fundamental to human cognition and behaviour. Everyday life often requires us to learn new skills, and effective instructions can greatly facilitate this process. Indeed, it is difficult to imagine how a person could acquire certain complex skills (such as driving a car) *without* explicit instructions. Inside the laboratory, participants in cognitive psychology experiments typically demonstrate an impressive ability to master novel and arbitrary choice reaction time tasks via instructions, often with very little practice. In comparison to non-human species, who can be taught simple cognitive tasks with great effort and after many months of practice (e.g., Nakahara et al., 2002), humans appear especially adept at rapidly assimilating unfamiliar instructions.

Research has shown that instructions can have a powerful effect on behaviour, and can result in the automatic activation of stimulus-response mappings, even without any practice. For example, Cohen-Kdoshay and Meiran (2009) used a variant of the flanker paradigm, in which participants were instructed to respond to a centrally presented target whilst ignoring adjacent “flankers”. Crucially, participants received new task instructions and new stimuli for each block. Cohen-Kdoshay and Meiran (2009) found a significant flanker effect even on the very first trial of each block, suggesting that flankers automatically activated the competing responses purely based on instructions (also see Meiran et al., 2017; Liefooghe & De Houwer, 2018). Although these results demonstrate that instructions can have a profound effect on behaviour, much less is known about the cognitive mechanisms that enable participants to proceduralise task instructions prior to performance.

One of the processes thought to be crucial to our ability to follow instructions is language. Recent theories of instruction following have argued that instruction following consists of three distinct phases: (1) An instruction phase, during which linguistic information is translated into a task model; (2) an implementation phase, during which the task model is implemented; and (3) the application phase, during which the relevant condition-action rule is applied automatically (Brass et al., 2009; Brass et al., 2017). According to this model, language is especially important during the first phase of learning. Theories of skill acquisition (Anderson, 1982), task-set control (Monsell, 2017) and working memory (Oberauer, 2009) similarly assume that although a declarative representation might aid the acquisition of a novel task, the execution of well-practiced tasks is ultimately dependent on a non-linguistic, procedural representation of that task.

In support of this assumption, van ’t Wout and Jarrold (2020) recently showed that language plays a crucial role in the acquisition of novel tasks via trial-and-error learning. Participants learned a series of novel tasks (each task consisting of five arbitrary stimulus-response rules) whilst performing a verbal distractor task (articulatory suppression), a non-verbal distractor task (foot tapping) or no distractor task. The results showed that participants made more errors under articulatory suppression (compared to foot tapping), but only at the beginning of each task. Once the task was well-practiced, articulatory suppression no longer had a detrimental effect on performance.

This detrimental effect of a verbal distractor task (articulatory suppression) demonstrates that language plays a crucial role in the acquisition of novel tasks via trial-and-error learning. However, few studies have investigated whether language is also used to encode instructions provided *before* a novel task. Data from a very recent study by Monsell and Graham (2021; Experiments 1 and 2) are consistent with this possibility. Following an instruction phase, participants performed a series of six-choice reaction time tasks (with six object pictures mapping onto six unique response keys) in which the stimulus names were phonologically either similar or dissimilar. These experiments showed that performance was worse for tasks comprised of phonologically similar stimuli, but that this effect diminished after just four presentations of each stimulus, suggesting that verbal mediation of task performance is extremely short-lived.

Although these results suggest that early performance during instruction-based learning is mediated by a verbal representation, it cannot be concluded with certainty that participants used language to *encode* the task instructions. Instead, it is also possible that phonological similarity was the driver of poorer recall *during* performance of the task. The current study sought to determine whether language is used to encode novel task instructions, by implementing a different manipulation (articulatory suppression), which was applied only during the instruction phase, and not during task performance itself. Specifically, in the two experiments reported here, participants were required to learn a series of choice reaction time tasks, with each task consisting of six picture stimuli (different pictures were used for each task), mapped arbitrarily onto six response keys. The instruction phase consisted of a visual representation of the correct stimulus-response rules, so that participants were not forced to rely on language to encode the instructions (as they would have been if the instructions had been presented verbally). Rather, participants were able to use either a linguistic or a non-linguistic strategy for encoding. During the instruction phase (but not during task performance itself) participants were required to perform articulatory suppression, foot tapping, or no distractor task. If proceduralisation of the task during the instruction phase is reliant on language, then performance should be worse under articulatory suppression than in the foot tapping or no distractor task conditions.

Additionally, both experiments also manipulated the instruction duration, which was either long (60 s in both experiments) or short (30 s in Experiment 1, and 10 s in Experiment 2). In both experiments participants performed each distractor task once under each of the instruction duration conditions. With regard to the effect of instruction duration, we predicted two possible outcomes: On the one hand, it is possible that with a long instruction duration, participants are able to effectively proceduralise the instructions even under articulatory suppression, resulting in a reduced effect of articulatory suppression with a long instruction duration relative to a short one. Alternatively, it is possible that the detrimental effect of articulatory suppression could be more pronounced with a long instruction duration, if participants in that condition were able to benefit more fully from the use of linguistic strategies under foot tapping.

## Experiment 1

### Participants

All 48 participants^1^ (see Table 1) received course credit in return for their participation, and provided informed consent prior to taking part. Experiment 1 was approved by the University of Bristol’s School of Psychological Science’s Human Research Ethics Committee (ID 104923).

**Table 1:** Participant demographic information from Experiments 1 and 2

	Participants	Mean age (minimum–maximum)	Female/male
Experiment 1	48	23 (18–36)	29/19
Experiment 2	48	26 (18–50)	32/16

Please note that data from 11 participants (six from Experiment 1 and five from Experiment 2) with mean error rates more than three standard deviations above the grand average were removed and replaced

### Method

The experiment required participants to complete six choice reaction time tasks. For each task, participants were required to respond to a set of six picture stimuli (presented one at a time) using six unique response keys.^2^ All six tasks were identically structured, but new picture stimuli were used in each task, so that participants had to learn a new set of six arbitrary S-R mappings for each task. Stimuli were line drawings selected from the International Picture Naming Project (IPNP; Bates et al., 2003). Six sets of six pictures each (plus one extra set for practice, not included in the analysis) were created (see Table 2). Each task contained 36 trials, so that every stimulus appeared six times within a task. The sequence of trials within a task was pseudorandomised to avoid immediate stimulus repetitions.

**Table 2:** Picture names for the stimulus sets used in Experiments 1 and 2

**#**	**Set 1**	**RT**	**%**	**#**	**Set 2**	**RT**	**%**	**#**	**Set 3**	**RT**	**%**
1	egg	874	98	7	spoon	777	100	13	bus	771	100
2	car	751	100	8	tent	744	100	14	leaf	848	100
3	tree	796	100	9	box	753	100	15	pen	753	100
4	fan	865	98	10	pig	855	100	16	house	745	98
5	sock	712	100	11	ear	681	100	17	dog	702	100
6	hat	684	98	12	watch	780	100	18	cake	789	100
	**Mean**	**780**	**99**		**Mean**	**765**	**100**		**Mean**	**768**	**100**
**#**	**Set 4**	**RT**	**%**	**#**	**Set 5**	**RT**	**%**	**#**	**Set 6**	**RT**	**%**
19	heart	720	100	25	frog	751	100	31	bed	706	100
20	owl	837	98	26	chair	732	100	32	fish	777	100
21	foot	758	98	27	hand	723	98	33	cheese	843	100
22	moon	804	100	28	train	838	100	34	clock	772	98
23	key	738	100	29	snake	775	100	35	dress	840	100
24	bread	773	98	30	kite	796	100	36	eye	700	98
	**Mean**	**771**	**99**		**Mean**	**769**	**100**		**Mean**	**773**	**99**

Stimuli were matched for percent name agreement (%) and average naming latency (ms; norms obtained from the IPNP). Images within a set were selected as to avoid phonological, semantic or visual similarity

**Fig. 1:**
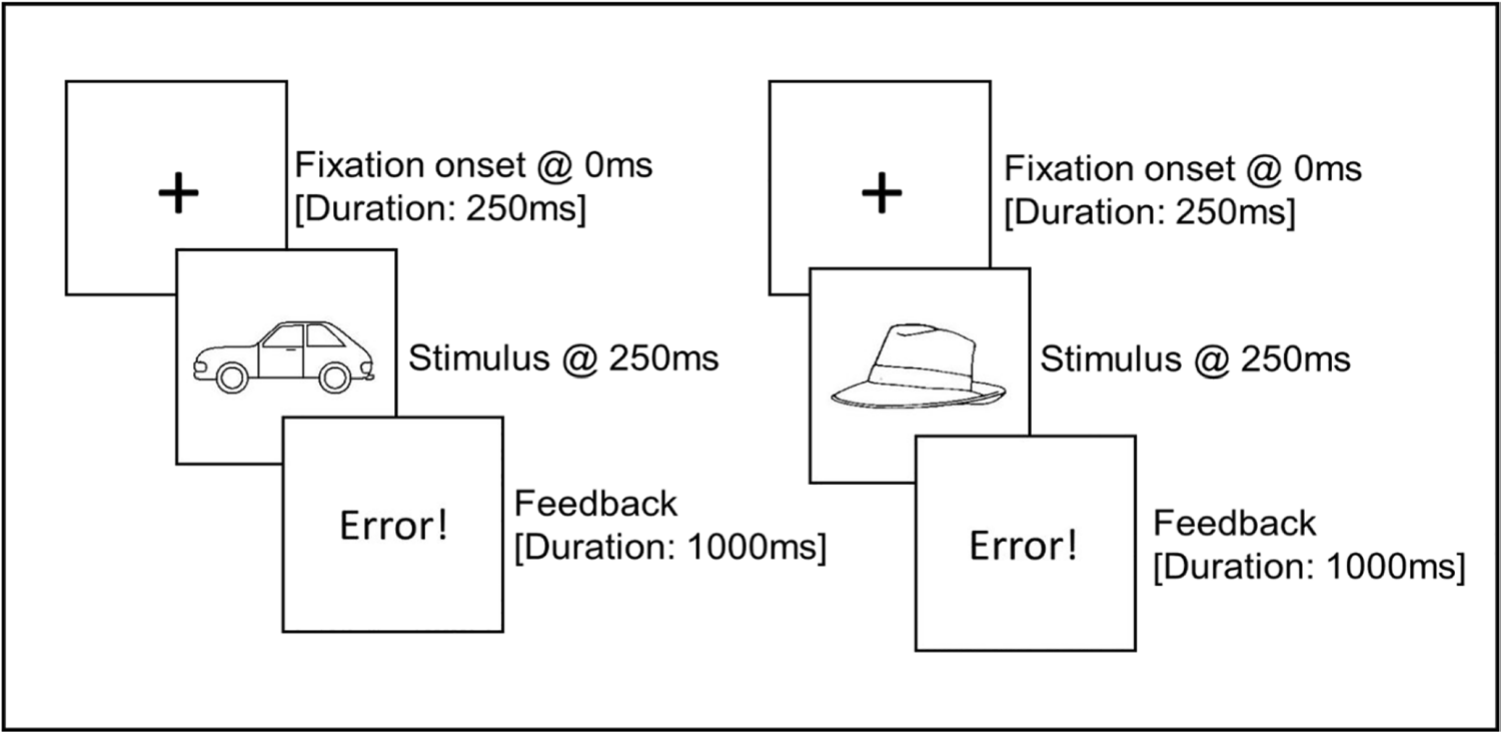
Example of a sequence of two consecutive trials. The trial sequence was identical in Experiments 1 and 2

Each task required participants to respond to a centrally presented target image with the x, c, v, b, n or m key on a standard QWERTY keyboard (using the ring, middle and index finger of both hands). Each trial consisted of a 250-ms fixation cross, followed by the stimulus, which remained on screen until a response was made. Feedback was provided on incorrect trials (see Fig. 1).

**Fig. 2:**
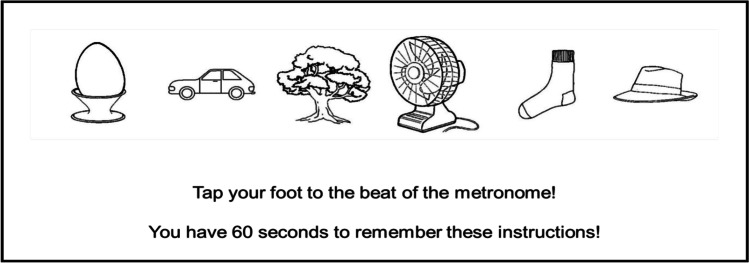
Example of an instruction screen displayed to participants (in the foot tapping condition; instruction duration 60 s) prior to the start of a task

Prior to the start of each task, participants were informed of the correct stimulus-response mappings for that task. Specifically, they viewed an instruction screen simultaneously displaying all six stimuli in that task, from left to right, in a serial order (i.e. with the stimulus mapping onto the x response in the leftmost position, and the stimulus mapping onto the m response in the rightmost position; see Fig. 2 for an example). For half the tasks, participants were informed they had 30 s to view the instruction screen. For the other half of the tasks, participants were informed they had 60 s to view the instruction screen.^3^

To investigate the role of language in instruction encoding, there were three distractor task conditions: For the duration of the instruction phase only (and not during task performance itself), participants were required to perform either articulatory suppression, foot tapping, or no distractor task. These distractor tasks were chosen as previous research has demonstrated that they are well matched in terms of difficulty (van ’t Wout & Jarrold, 2020). Articulatory suppression (saying “tick, tick, tick”) and foot tapping (tapping one foot) were performed to a metronome set to beat at 100 beats per minute. During the no distractor task condition, the metronome played, but participants were instructed to ignore it. The order of distractor task conditions and instruction duration conditions was balanced between subjects, as was the assignment of stimuli to conditions and the assignment of stimuli to responses. In this way, each participant completed one task for each combination of the distractor task and instruction duration conditions (six tasks in total).

Prior to completing these six tasks, participants first practised foot tapping and articulatory suppression for 60 s each, after which they performed one practice task of 36 trials (identical to the no distractor task condition, and with a 60-s instruction duration).

The experiment was programmed in PsychoPy (Peirce et al., 2019) and run online,^4^ via Pavlovia. All participants were instructed to complete the experiment in a quiet space, on a computer or laptop with access to internet and sound. In total, the experiment lasted 20 min, after which participants were debriefed via e-mail.

### Results

To examine the effects of instruction duration and distractor task type on performance, two separate 2 (instruction duration: 30s or 60s) x 3 (distractor task type: articulatory suppression, foot tapping or no distractor task) repeated-measures ANOVAs were run on the % error data and the mean correct RT data. Prior to data analysis, RTs smaller than 200 ms or greater than 5,000 ms (0.9% of correct responses) were removed from the data set.

**Fig. 3:**
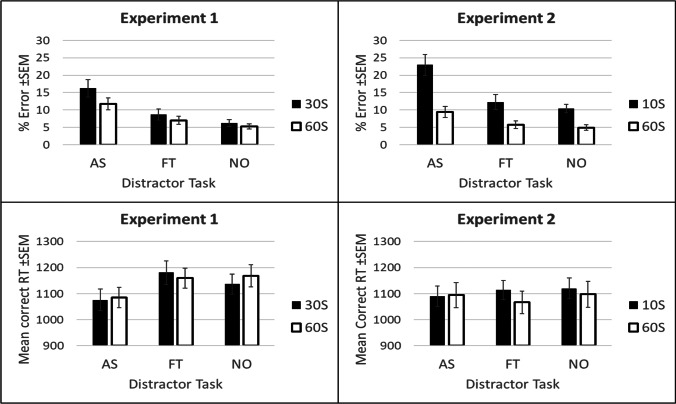
(Top) % Error data and (bottom) mean correct reaction time (RT) data from Experiments 1 and 2, plotted as a function of distractor task condition (articulatory suppression (AS), foot tapping (FT) or none) and instruction screen duration (60 s or 10/30 s)

Analysis of the error data produced a significant main effect of instruction duration: Error rates were greater with a shorter 30-s instruction duration (10.4 ± 1.1%^5^) than with a longer 60-s instruction duration (8.0 ± 0.8%), F(1,47) = 4.70, p = .035, ${\eta}_p^2$= .091 (see Fig. 3). The main effect of distractor task type was also significant, F(2,94) = 13.44, p < .001, ${\eta}_p^2$= .222 (Huynh-Feldt; H-F). Three further one-way ANOVAs compared the data from each distractor task condition (pooled across instruction duration) against one another. Error rates were increased under articulatory suppression (14.0 ± 1.7%) compared to foot tapping (7.8 ± 1.1%), F(1,47) = 10.45, p = .002, ${\eta}_p^2$= .182; and increased under articulatory suppression compared to the no distractor task condition (5.8 ± 0.7%), F(1,47) = 20.99, p < .001, ${\eta}_p^2$= .309. Error rates were marginally increased under foot tapping compared to the no distractor task condition, F(1,47) = 3.47, p = .069, ${\eta}_p^2$= .069. Although the effect of distractor task type was numerically larger in the 30-s (articulatory suppression - foot tapping difference: 7.6 ± 2.9%) than in the 60-s condition (articulatory suppression - foot tapping difference: 4.8 ± 1.7%), the interaction between instruction duration and distractor task type was not significant, F(2,94) = 0.97, p = .373, ${\eta}_p^2$= .020 (H-F).

For the mean correct RT data, the same 2 (instruction duration: 30 s or 60 s) x 3 (distractor task type: articulatory suppression, foot tapping or no distractor task) repeated-measures ANOVA revealed only a significant main effect of distractor task type, F(2,94) = 8.77, p < .001, ${\eta}_p^2$= .157 (H-F); the main effect of instruction duration, F(1,47) = 1.24, p = .726, ${\eta}_p^2$= .003, and the interaction between instruction duration and distractor task type, F(2,94) = 0.64, p = .518, ${\eta}_p^2$= .014, were not significant. Three further one-way ANOVAs pooled across instruction duration compared the mean RT in each distractor task condition. These analyses revealed that RTs were faster under articulatory suppression (1,081 ± 36ms) than under foot tapping (1,170 ± 39ms), F(1,47) = 17.44, p < .001, ${\eta}_p^2$= .271; and faster under articulatory suppression than in the no distractor task condition (1,153 ± 37ms), F(1,47) = 7.36, p = .009, ${\eta}_p^2$= .135 (see Fig. 3). The difference between foot tapping and the no distractor task condition was not significant, F(1,47) = 0.62, p = .434, ${\eta}_p^2$= .013.

Increased error rates and faster RTs in the articulatory suppression condition suggest the possibility of a speed-accuracy trade-off. To examine whether the increased error rates under articulatory suppression compared to foot tapping were caused exclusively by faster RTs in the articulatory suppression condition, an additional correlational analysis was conducted on the articulatory suppression - foot tapping difference for the error rates and mean correct RTs (averaged across instruction duration). This analysis revealed no significant correlation between the articulatory suppression - foot tapping difference for error rates and mean correct RTs, r(48) = -.20, p = .182, suggesting that the detrimental effect of articulatory suppression on accuracy is not exclusively the result of a speed-accuracy trade-off.

Finally, an exploratory analysis examined whether the effects of distractor task and instruction duration varied over time within a task. First, a pair of one-way repeated-measures ANOVAs examined the effect of trial number (1–36) on mean correct RT and % error data (pooled across conditions). These analyses showed that mean correct RT and % error decreased linearly as a function of trial number (RTs: slope -7 ± 1 ms per trial number, linear trend: F(1,47) = 37.99, p < .001, ${\eta}_p^2$= .447; % error: slope -0.1 + 0.0% per trial number, linear trend: F(1,47) = 13.13, p < .001, ${\eta}_p^2$= .218). To examine whether this decrease in RT and error rate with trial number was modulated by condition, a pair of 2 (distractor task type: articulatory suppression or foot tapping) x 2 (instruction duration: 30 s or 60 s) repeated-measures ANOVAs were conducted on the individual linear slopes for the % error and correct RT data. These ANOVAs yielded no significant main effects or interactions (all Fs < 2.63), suggesting that improvements in performance with practice were not significantly modulated by distractor task or instruction duration.

## Summary

Experiment 1 investigated whether performance on a novel task was affected by the distractor task performed during the instruction phase. It also manipulated the duration of the instruction phase, which was either 30 s or 60 s. As expected, error rates were greatest when participants had performed articulatory suppression during the instruction phase, suggesting that language plays a crucial role in instruction encoding. This detrimental effect of articulatory suppression was not significantly modulated by instruction duration (though see Experiment 2). Furthermore, an exploratory analysis examining the effect of practice found that although RTs and error rates decreased throughout each task, these improvements in performance with practice were not significantly modulated by distractor task or instruction duration. Finally, RTs were also faster under articulatory suppression than in the foot tapping condition and the no distractor task condition, a pattern that has been reported in some previous studies (Bryck & Mayr, 2005; Weywadt & Butler, 2013). Importantly, an additional correlational analysis demonstrated that the detrimental effect of articulatory suppression on accuracy cannot be exclusively attributed to a speed-accuracy trade-off. Furthermore, as this RT result was not replicated in Experiment 2, we do not dwell on this finding.

## Experiment 2

Experiment 2 further explored the effect of instruction duration and distractor task type on performance. Specifically, it is possible that the interaction between instruction duration and distractor task type was not significant in Experiment 1 because the 30-s instruction interval did not induce sufficient time pressure, meaning that participants were still able to verbally encode the instructions to some extent, even with an instruction duration of 30 s. Therefore, Experiment 2 used a 10-s and a 60-s instruction duration interval. In all other ways, the design of Experiment 2 was identical to that of Experiment 1.

### Participants

All 48 participants (see Table 1) provided informed consent prior to taking part, and received a £4 Amazon voucher in return for their participation. Experiment 2 was approved by the University of Exeter’s Psychology Ethics Committee (ID eCLESPsy002328).

### Results

To examine the effects of instruction duration and distractor task type on performance, separate 2 (instruction duration: 10 s or 60 s) x 3 (distractor task type: AS, foot tapping or no distractor task) repeated-measures ANOVAs were run on the % error data and the mean correct RT data. Prior to data analysis, RTs smaller than 200 ms or greater than 5,000 ms (0.7% of correct responses) were removed from the data set.

Analysis of the error data produced a significant main effect of instruction duration, F(1,47) = 25.16, p < .001, ${\eta}_p^2$= .349; a significant main effect of distractor task type, F(2,94) = 16.74, p < .001, ${\eta}_p^2$= .263 (H-F; see Fig. 3); and a significant interaction between instruction duration and distractor task type, F(2,94) = 5.86, p = .004, ${\eta}_p^2$= .111 (H-F). Critically, a further two-way ANOVA excluding the no distractor task condition found that the detrimental effect of articulatory suppression compared to foot tapping was significantly greater in the 10-s instruction duration condition (articulatory suppression - foot tapping difference: 10.7 ± 2.6%) compared to the 60-s instruction duration condition (articulatory suppression - foot tapping difference: 3.7 ± 1.3%), F(1,47) = 7.86, p = .007, ${\eta}_p^2$= .143. Further one-way ANOVAs comparing each distractor task condition for each instruction duration condition separately found that for both instruction durations, participants made significantly more errors under articulatory suppression than foot tapping (10 s: F(1,47) = 16.99, p < .001, ${\eta}_p^2$= .265; 60 s: F(1,47) = 8.05, p = .007, ${\eta}_p^2$= .146); and under articulatory suppression than in the no distractor task type condition (10 s: F(1,47) = 17.94, p < .001, ${\eta}_p^2$= .276; 60 s: F(1,47) = 8.97, p = .004, ${\eta}_p^2$= .160). These results show that a detrimental effect of articulatory suppression was found even with a long instruction duration. The difference between foot tapping and the no distractor task type condition was not significant in either instruction duration condition (10 s: F(1,47) = 0.77, p = .384, ${\eta}_p^2$= .016; 60 s: F(1,47) = 0.61, p = .439, ${\eta}_p^2$= .013).

For the mean correct RT analysis, the same 2 (instruction duration) x 3 (distractor task type) repeated-measures ANOVA showed that both the main effects of instruction type and distractor task type were not significant (F(1,47) = 0.89, p = .349, ${\eta}_p^2$= .019 and F(1,47) = 0.34, p = .680, ${\eta}_p^2$= .007 (H-F), respectively). The interaction between instruction duration and distractor task type was also not significant, F(1,47) = 1.09, p = .342, ${\eta}_p^2$= .023 (H-F).

Finally, in line with Experiment 1, an additional exploratory analysis examined whether the effects of distractor task type and instruction duration were modulated by practice. As for Experiment 1, a pair of one-way repeated- measures ANOVAs examining mean correct RT and error rates as a function of a trial number (1–36) showed that mean correct RTs and error rates decreased linearly as a function of trial number (RTs: slope -10 ± 1ms per trial number, linear trend: F(1,47) = 156.02, p < .001, ${\eta}_p^2$ = .768; % error: slope -0.2 + 0.0% per trial number, linear trend: F(1,47) = 26.39, p < .001, ${\eta}_p^2$ = .360). To examine whether this decrease in RT and error rate with trial number was modulated by condition, a pair of 2 (distractor task type: articulatory suppression or foot tapping) x 2 (instruction duration: 10 s or 60 s) repeated-measures ANOVAs were conducted on the individual linear slopes for the RT and % error data. The RT analysis yielded no significant main effects or interactions (all Fs < 1.67). The analysis of error rates yielded significant main effects of instruction duration, F(1,47) = 8.88, p = .005, ${\eta}_p^2$ = .159, and distractor task type, F(1,47) = 4.20, p = .046, ${\eta}_p^2$ = .082, but the two-way interaction was not significant, F(1,47) = 1.41, p = .242, ${\eta}_p^2$ = .029. The significant main effects of instruction duration and distractor task type reflected that the decrease in error rates as a function of trial number was greater in the short instruction duration condition (slope -0.4 + 0.1% per trial number) compared to the long instruction duration condition (slope -0.1 + 0.1% per trial number), and greater in the articulatory suppression condition (slope -0.3 + 0.1% per trial number) than in the foot tapping condition (slope -0.1 + 0.1% per trial number).

### Summary

Experiment 2 investigated whether, with a shorter instruction duration of 10 s, the detrimental effect of articulatory suppression would vary as a function of instruction duration. This prediction was confirmed, as the difference between articulatory suppression and foot tapping was significantly increased with the shorter instruction duration of 10 s compared to the longer instruction duration of 60 s. Furthermore, Experiment 2 did not replicate the faster RTs in the articulatory suppression condition (compared to the foot tapping and no distractor task conditions) found in Experiment 1. Finally, an exploratory analysis examining the effects of practice found that error rates and RTs decreased throughout each task, and that the improvement in accuracy with practice was greater in the articulatory suppression condition than in the foot tapping condition, and greater with a short instruction duration than with a long instruction duration.

### Discussion

The two experiments reported here investigated whether participants use language to encode novel task instructions. Both experiments unequivocally supported that possibility, by demonstrating that participants made more errors when articulatory suppression had been performed during the instruction phase, compared to foot tapping. Experiment 2 furthermore found that when the instruction interval was short (10 s), the detrimental effect of articulatory suppression was larger than when the instruction interval was long (60 s).

One of the predicted outcomes was that the effect of articulatory suppression might be more pronounced with a short instruction duration, as with a long instruction duration participants may be able to encode the instructions even under articulatory suppression. In line with this prediction the effect of articulatory suppression was significantly greater with a short instruction duration (Experiment 2). However, in both experiments participants still made significantly more errors under articulatory suppression than foot tapping with a long instruction duration. There are two possible explanations for this latter finding: First, it is possible that language is the most efficient and effective strategy for instruction encoding, and that a non-verbal strategy (which participants might be forced to adopt under articulatory suppression) is less successful, resulting in more errors under articulatory suppression, even when plenty of time (60 s) is available for encoding. Another possible explanation is that articulatory suppression did not block attempts at phonological recoding completely. This latter suggestion is consistent with a number of studies which have shown that participants are still able to perform phonological judgments (e.g. judge whether pairs of words are rhymes or homophones), even when they are performing articulatory suppression (see Norris, Butterfield, Hall, & Page, 2018). Hence, in the current experiments, it is possible that participants still engaged in phonological recoding under articulatory suppression, but to a lesser or less efficient extent than in the other two distractor conditions. Either way, the significant interaction between instruction duration and distractor task in Experiment 2 shows that articulatory suppression especially affects instruction encoding under time pressure.

The detrimental effect of articulatory suppression on accuracy clearly demonstrates that participants attempted phonological recoding during the instruction phase. These experiments therefore provide the first conclusive evidence that language plays a crucial role in in encoding task instructions, as suggested by theories of instruction following (Brass et al., 2017). When the use of phonological recoding is disrupted, subsequent task performance evidently suffered. One might ask *why* participants engaged in phonological recoding at all, given that the task did not strictly depend on it (both the instructions and the stimuli used were visual, and the task could arguably have been performed by relying on visual strategies instead). One explanation as to why participants might have still attempted phonological recoding in the current experiments is that the capacity of verbal short-term memory (± 7 items when rehearsal is available^6^; Miller, 1956) is thought to exceed the capacity of visual short-term memory. Research suggests that the latter holds no more than three chunks (Zhang & Simon, 1985) or four objects (Luck & Vogel, 1997). As the current experiments required participants to encode six S-R rules, the task may have exceeded the capacity of visual short-term memory. The superior capacity of verbal (compared to visual) short-term memory may partly be why language is such a powerful tool when it comes to encoding instructions.

Finally, the current results are consistent with and extend those of Monsell and Graham (2021), who found that early performance in an instruction-based choice RT task is affected by the phonological similarity of the stimulus names. It is worth noting that Monsell and Graham (2021; Experiment 2) also included an exploratory manipulation of instruction duration (participants had either 10 or 14.5 s to encode the instructions). However, in contrast with our results from Experiment 2 (but in line with our results from Experiment 1), they did not find a significant interaction between phonological similarity and instruction duration, likely because the difference between their instruction durations was not pronounced enough.

With regard to the precise nature of the role of language in instruction encoding, a number of questions remain unanswered: Does the role of language in instruction encoding depend on the stimulus materials used? Is the use of verbal and non-verbal strategies in instruction encoding under strategic control (cf. Campoy & Baddeley, 2008)? Are children and adults with language difficulties able to compensate for such difficulties by effectively applying non-verbal strategies? Future research must attempt to answer these questions. Nevertheless, the current study has demonstrated that language plays a powerful role in the encoding of task instructions.

## Footnotes

1 The sample size was based on Brysbaert and Stevens (2018) recommendation of at least 1,600 observations per condition (trials x participants).

2 This number (six) of S-R mappings was chosen based on previous research, which found that when participants are required to learn sets of unfamiliar S-R mappings by trial-and-error, articulatory suppression had a detrimental effect on performance (compared to foot tapping), but only when the task had six S-R mappings, and not when it had three or four S-R mappings (van ‘t Wout & Jarrold, 2022).

3 The 60-s instruction duration was chosen based on data from a previous unpublished study (which employed a similar design), in which participants were able to view the instructions for as long as they liked. In that study, participants viewed each instruction screen for an average of 60 s.

4 Because this was an online experiment, we acknowledge that there was no way to ensure that participants were performing the distractor tasks as required. However, as the data revealed a very similar pattern of results compared to previous similar lab-based experiments (see van ’t Wout & Jarrold, 2020), it seems likely that the majority of the participants did perform the distractor tasks as requested.

5 Throughout, the number following the ± symbol indicates the standard error of the mean (SEM).

6 Note that the “pure” estimate of short-term memory when rehearsal is prevented is thought to be restricted to four chunks (Cowan, 2004).
